# Identification of Potential Candidate Dwarf Genes in Maize by BSA-Seq and Transcriptomic Analyses

**DOI:** 10.3390/plants15111715

**Published:** 2026-06-01

**Authors:** Lei Zhang, Yumei Zhou, Zelong Zhuang, Jianwen Bian, Xiaojia Hao, Zhenping Ren, Wanling Ta, Yunling Peng

**Affiliations:** 1 College of Agronomy, Gansu Agricultural University, Lanzhou 730070, China; 18919106150@163.com (L.Z.); 18793107830@163.com (Y.Z.); zhuangzl3314@163.com (Z.Z.); bjwen1018@163.com (J.B.); haoxj772@163.com (X.H.); renzp1003@163.com (Z.R.); kellytwl@163.com (W.T.); 2 Gansu Provincial Key Laboratory of Aridland Crop Science, Gansu Agricultural University, Lanzhou 730070, China; 3 Seed Industry Research Institute of Gansu Provincial University, Lanzhou 730070, China

**Keywords:** maize, dwarf mutant, bulked segregant analysis (BSA), RNA sequencing (RNA-seq), preliminary gene mapping

## Abstract

Maize (*Zea mays* L.) is a globally important crop. Understanding plant height and the underlying dwarfing genes is of critical importance for improving lodging resistance and optimizing plant architecture. In this study, a dwarf mutant designated CM17-2 was obtained by exposing the maize inbred line B73 to carbon ion beam irradiation. The mutant exhibited markedly reduced plant height, internode length, and ear height. Segregation analysis in the F_2_ population revealed a 3:1 ratio, indicating monogenic recessive inheritance. Using bulked segregant analysis (BSA), the causal locus was mapped to four intervals on chromosomes 2, 4, and 5. Exogenous GA_3_ treatment rescued the dwarf phenotype and restored plant height to near-wild-type levels, indicating GA sensitivity. Transcriptomic analysis following GA_3_ treatment identified 3292 differentially expressed genes (DEGs) between CM17-2 and B73. Among these, 1664 GA_3_-responsive genes were significantly enriched in the plant hormone signal transduction pathway. Integration of BSA-seq and transcriptomic data pinpointed 18 candidate genes on chromosome 4. Through gene annotation, four potential candidate genes likely associated with dwarfism were identified, and the functions of these candidate genes need to be validated in future experiments. The identification of these potential candidate genes lays a theoretical foundation for the subsequent functional analysis, gene cloning, and mechanistic studies of maize dwarfism-related genes.

## 1. Introduction

Maize (*Zea mays* L.), an annual herbaceous species within the Poaceae family, serves multifunctionally as a vital food crop, livestock feedstock, bioenergy feedstock, and industrial feedstock. Despite its agricultural significance, intensified weather extremes in recent years have impeded maize production, with lodging emerging as a primary yield-limiting factor [1,2,3,4]. Developing elite dwarf and lodging-resistant germplasm for widespread adoption constitutes a pivotal strategy to mitigate this challenge.

Plant height, a pivotal agronomic trait defining plant architecture, directly impacts planting density, lodging resistance, land-use efficiency, and economic yield in dwarf or compact maize cultivars [5]. Optimal plant height is crucial for stable and high yields. Stem node number and internode length determine maize height [6], with plant height reduction attributed to monogenic inheritance of qualitative traits and polygenic control of quantitative traits in crops [7,8,9].

Plant dwarfism arises from diverse etiologies, including internode shortening, decreased internode number, and aberrations in cell wall architecture or cell elongation [10]. To date, phenotypic characterization of plant dwarf mutants has predominantly implicated genes regulating the biosynthesis and signaling of gibberellin (GA), indole-3-acetic acid (IAA), brassinosteroids (BRs), and other phytohormones [11,12]. The rice semi-dwarf mutant sd1, which ignited the Green Revolution, manifests dwarfism due to a mutation in *SD1* (*semi-dwarf 1*), encoding GA 20-oxidase, an enzyme pivotal in gibberellin (GA) biosynthesis [13]. Furthermore, maize dwarf mutants *dwarf1* (*d1*), *dwarf3* (*d3*), *dwarf5* (*d5*), and *anther ear1* (*an1*) all result from mutations in genes encoding enzymes pivotal to the gibberellin (GA) biosynthesis pathway, which disrupt GA accumulation and induce the dwarf phenotype [14,15,16,17]. IAA plays a central role in modulating plant growth and development by regulating cell division and cell expansion processes [18]. Mutations in Arabidopsis *YUC* genes result in loss-of-function of a pivotal enzyme in the IAA biosynthesis pathway, thereby attenuating IAA production and causing phenotypes such as compromised apical dominance and diminished plant stature [19]. Despite their low abundance in plants, brassinosteroids (BRs) profoundly regulate plant dwarfism. Mutations in BR biosynthetic genes *smt1* and *smt2* impair signaling, leading to elevated endogenous BR levels, compromised vascular development, and dwarf architecture [20,21]. Conversely, the *Arabidopsis* BR-deficient mutant *det2* manifests dwarfism due to a loss-of-function mutation in *DET2*, which abolishes enzymatic activity and diminishes cell size [22].

Recently, molecular biology advancements have facilitated the discovery and characterization of numerous crop dwarfing genes [23,24]. Plant dwarfing breeding originated in the early 20th century when British researchers first isolated a height-regulating allele effective across diverse plant species. Dwarf mutants constitute a crucial reservoir of dwarfing traits for crop breeding [25]. The introgression of dwarf and semi-dwarf alleles has been demonstrated to significantly boost lodging resistance and grain yield in maize. During the seminal Green Revolution, breeders engineered plant architecture through semi-dwarf trait introgression, culminating in high-performance cultivars with superior lodging resilience. The integration of sophisticated agronomic strategies with these genetic advancements engendered a substantial enhancement of maize productivity during the seminal Green Revolution [26]. Dissecting the genetic basis of plant height and deciphering regulatory mechanisms in maize have remained paramount objectives in modern breeding and fundamental research. To date, the Maize GDB Phenotype Database [27] catalogs comprehensive records of diverse dwarf germplasm resources. Over 60 maize dwarfing loci have been characterized; however, pleiotropic constraints posed by unfavorable allelic associations with deleterious traits hinder their translation into elite cultivar development [28,29,30]. Notwithstanding, limited progress has been achieved in developing and disseminating dwarf hybrid cultivars, which exhibit constrained germplasm diversity [29]. Consequently, deciphering novel allelic variants regulating plant height in maize is imperative for driving productivity advancements.

In this study, an F_2_ population was generated by self-pollinating the F_1_ hybrid derived from a cross between the dwarf mutant CM17-2 and its wild-type parent B73. By analyzing the phenotypes of the mutant, the F_2_ population, and employing bulked segregant analysis (BSA) combined with transcriptome sequencing, we performed preliminary mapping of the causal mutation. This work lays a theoretical foundation for identifying dwarfism-related genes and elucidating the molecular mechanisms underlying maize dwarfing.

## 2. Results

### 2.1. Acquisition and Genetic Analysis of Dwarf Mutants

The dwarf mutant CM17-2 was generated from the maize inbred line B73 via carbon ion beam mutagenesis. Briefly, seeds of B73 were irradiated with 80 Gy of carbon ion beams at the Institute of Modern Physics, Chinese Academy of Sciences. Following germination, 500 seedlings were cultivated to flowering, and 300 plants were self-pollinated. Seeds harvested from each selfed plant were sown in the greenhouse of the maize research group at Gansu Agricultural University. Seedlings of the resulting CM17 family exhibited segregation for plant height, and a single dwarf plant, presumed to carry the dwarf mutation, was selected. This plant was self-pollinated by artificial bagging, and seeds harvested at maturity were designated as the M_1_ generation. M_1_ seeds were planted as ear-to-row rows to evaluate plant height at both seedling and adult stages. From these rows, one individual displaying the most pronounced dwarf phenotype, vigorous growth, compact plant architecture, and superior agronomic traits was selected, bagged, and self-pollinated to produce M_2_ seeds. This selection and selfing procedure was repeated through the M_3_, M_4_, and M_5_ generations. After five consecutive generations of selfing, the dwarf trait was completely fixed; all plants within the line showed uniform plant height without further segregation, yielding an inbred line that was phenotypically uniform in both dwarf stature and key agronomic characteristics. This homozygous dwarf inbred line was subsequently designated CM17-2.

Both CM17-2 and B73 were planted at the Experimental Base of Gansu Agricultural University. At anthesis, pollen from CM17-2 was manually applied to the silks of B73 to produce F_1_ hybrid progeny. The F_1_ population was grown at the Experimental Station of Gansu Agricultural University in the following growing season and self-pollinated to generate an F_2_ segregating population. A total of 300 F_2_ seeds were grown in a controlled-environment growth chamber to determine the phenotypic segregation ratio. Phenotypic analysis revealed a 3:1 segregation ratio of tall to dwarf plants (Table 1), consistent with Mendelian monogenic recessive inheritance, thereby confirming the stable heritability of the dwarf trait.

### 2.2. Phenotypic Characterization of the Dwarf Mutant

**Figure 1:**
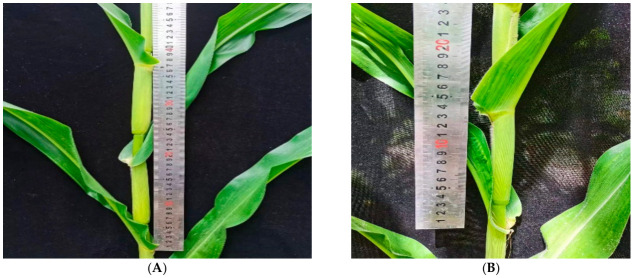
Phenotype of the wild type and mutant. Note: (**A**): wild type B73; (**B**): mutant CM17-2.

As shown in Figure 1 and Table 2 (*t*-test, *p* < 0.05), the plant height and internode length of the dwarf mutant CM17-2 were significantly reduced compared to the wild-type B73. Moreover, CM17-2 exhibited highly significant differences from B73 in plant height, internode length, and ear height, and a significant difference in stem diameter. Specifically, the plant height, internode length, and ear height of CM17-2 were reduced by 41.35%, 27.84%, and 44.24%, respectively, compared to B73, while its stem diameter increased by 7.85% relative to B73.

### 2.3. Initial Genetic Mapping of the Dwarf Mutant in Maize

#### 2.3.1. Initial BSA-Based Mapping of the CM17-2 Dwarf Mutant

**Figure 2:**
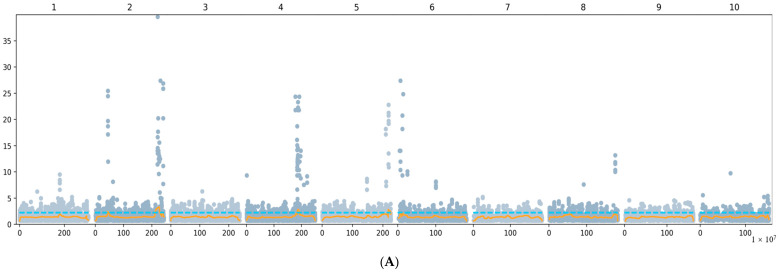

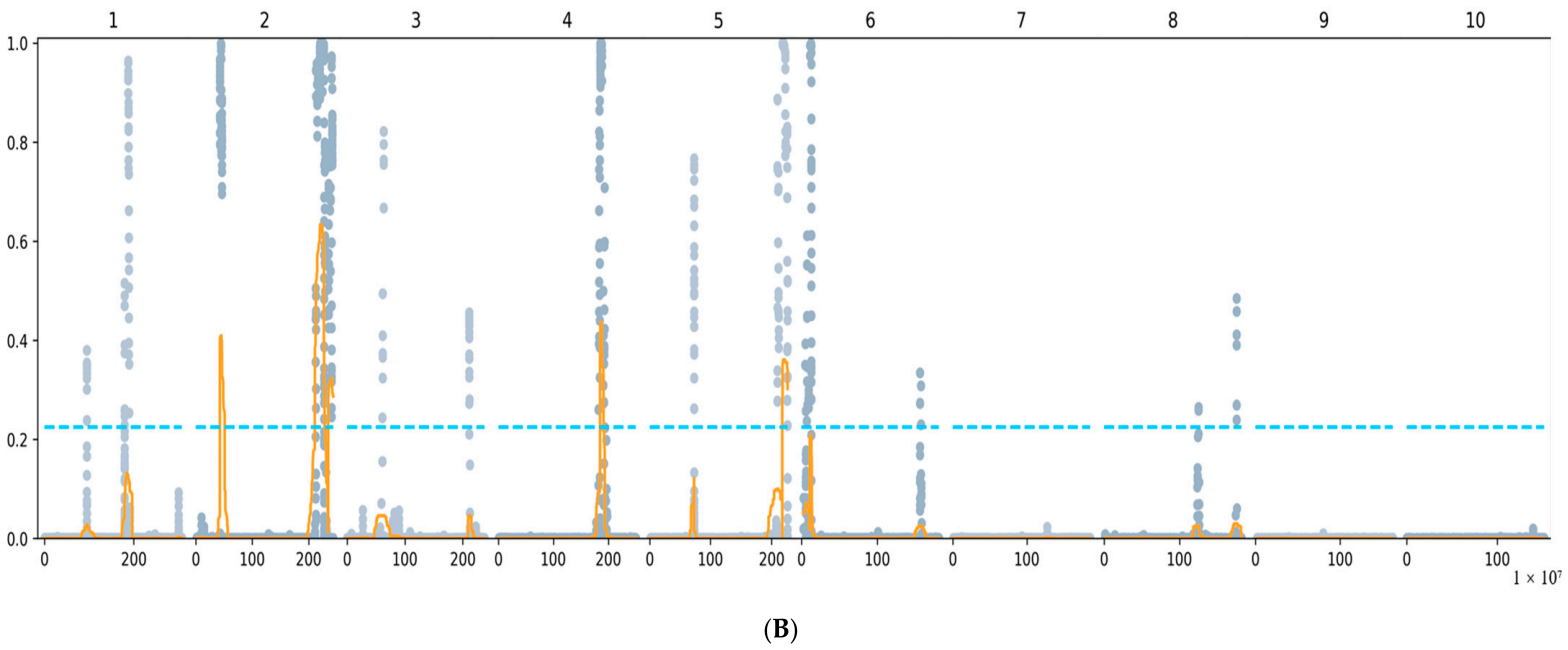
Primary gene localization in maize dwarf mutants CM17-2. Note: (**A**) is the 0-Ridit algorithm; (**B**) is the 0-DL algorithm.

A segregating F_2_ population derived from the dwarf mutant CM17-2 was established at the Experimental Base of Gansu Agricultural University. DNA bulking and sequencing were performed in accordance with BSA requirements. Identified SNPs were stringently filtered to retain high-confidence polymorphic loci, which were subjected to 0-DL and 0-Ridit algorithms to delimit candidate genomic regions. Both algorithms yielded largely consistent results, as demonstrated in Figure 2. Two non-overlapping candidate intervals on chromosome 2 were resolved: one spanning 15.95 Mb (212.10–228.05 Mb) and the other 3.36 Mb (42.79–46.15 Mb). An additional interval was mapped to chromosome 4 (7.15 Mb; 184.94–192.09 Mb) and chromosome 5 (8.72 Mb; 217.60–226.32 Mb).

#### 2.3.2. Candidate Gene Screening and Annotation

Mapping analysis of the F_2_ BSA data identified a candidate locus on chromosome 4 spanning 185.00–192.29 Mb, within which we hypothesize that the key gene controlling plant height resides. By querying the MaizeGDB database (https://www.maizegdb.org/, accessed on 3 November 2025) for functional annotations within this interval and excluding loci lacking functional descriptions or annotation records, a total of 113 candidate genes potentially associated with dwarf stature expression were identified (Supplementary Table S1).

### 2.4. Physiological Response of the Maize Dwarf Mutant to GA_3_

#### 2.4.1. Morphological Analysis of Maize Inbred Lines Under Exogenous Substance Treatment

**Figure 3:**
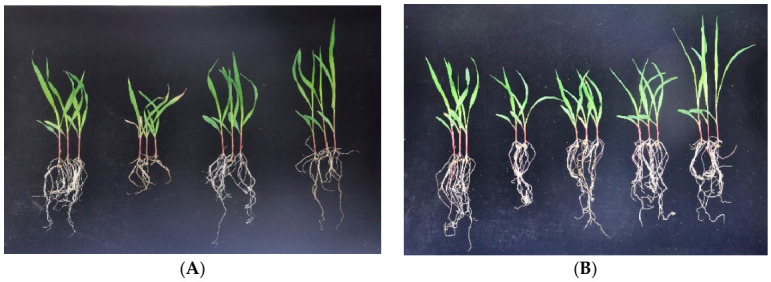
Phenotypes of wild-type B73 and dwarf mutant F_2_. Note: (**A**): Changes in wild-type B73 under different hormones, from left to right: CK_B73_, IAA_B73_, BL_B73_, GAB73; (**B**): Changes in mutant CM17-2 under different hormones, from left to right, CK_B73_, CK_CM17-2_, IAA_CM17-2_, BL_CM17-2_, GA_CM17-2_.

To elucidate the hormonal response mechanisms of the maize dwarf mutant, exogenous brassinolide (BL), auxin (IAA), and gibberellin (GA_3_) were applied to maize seedlings. As depicted in Figure 3, exogenous application of IAA, BL, or GA_3_ to wild-type B73 elicited significant elongation of both seedling and internode lengths, with GA_3_ treatment inducing the most pronounced elongation (*p* < 0.05). Similarly, treatment of the CM17-2 F_2_ dwarf mutants with IAA, BL, or GA_3_ resulted in marked increases in seedling and internode lengths compared to the control. Notably, GA_3_ treatment elicited significantly greater elongation than BL or IAA treatments, indicating enhanced sensitivity of the dwarf mutant to GA_3_ signaling.

**Figure 4:**
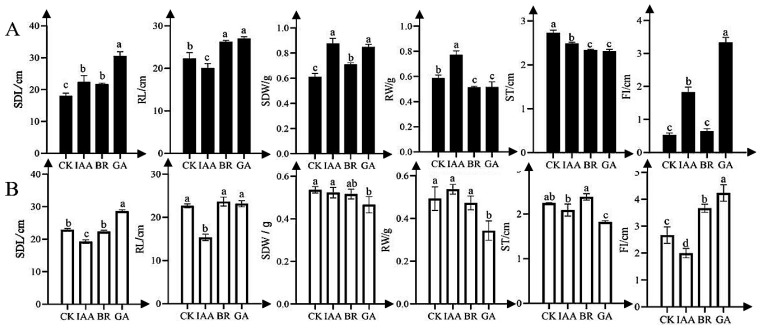
Phenotypic changes in wild-type B73 and dwarf mutants under treatment with exogenous substances. Note: (**A**): phenotypic changes of CM17-2 under different treatments; (**B**): phenotypic changes of B73 under different treatments; SDL: Seedling length; RL: Root length; SDW: Seedling fresh weight; RW: Root fresh weight; ST: Stem thickness; FI: First internode. Means ± standard errors are indicated within the bar graphs, and different lowercase letters represent significance at the 5% level.

As depicted in Figure 4, GA_3_ treatment elicited significant elevations in seedling and internode lengths in CM17-2 compared to the control (CK) (*p* < 0.05). Under IAA treatment, marked increases in seedling length, fresh above-ground biomass, dry above-ground biomass, and internode length were observed relative to CK (all *p* < 0.05). While brassinolide (BL) treatment enhanced seedling length, root elongation, fresh above-ground biomass, and internode length versus CK, the internode elongation did not reach statistical significance. Collectively, these data indicate that the CM17-2 dwarf mutant demonstrates enhanced responsiveness to GA_3_ in regulating the dwarf phenotype.

#### 2.4.2. Observation of Paraffin Sections of Maize Internodes Under GA_3_ Treatment

**Figure 5:**
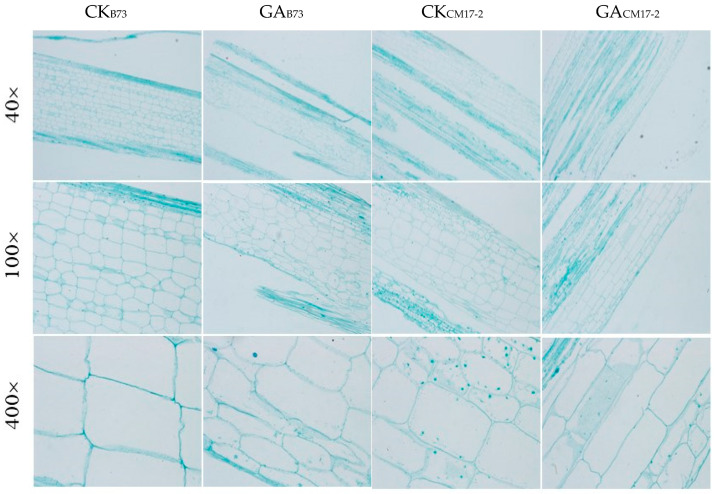
Longitudinal section of the first internode of the stem.

Longitudinal sections of GA_3_-treated maize internodes revealed that the dwarf mutant CM17-2 exhibited significantly shorter cell lengths and a reduced cell number compared to wild-type B73. Exogenous GA_3_ treatment promoted internode elongation in CM17-2 through increased cell proliferation and lengthening, accompanied by decreased cell width resulting in attenuated, elongated cell morphologies (Figure 5).

**Figure 6:**
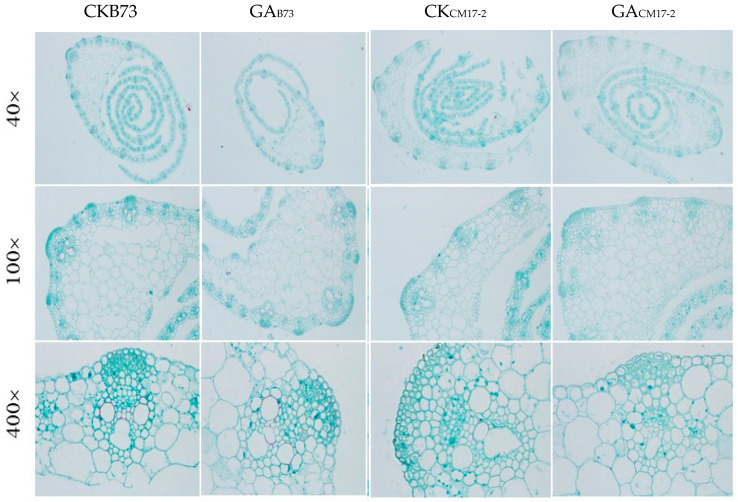
Cross-sectional view of the first internode of the stem.

Transverse sections of GA_3_-treated maize internodes revealed that the dwarf mutant CM17-2 exhibited a significantly elevated vascular bundle count compared to wild-type B73. This increase in vascular bundles, which enhance lodging resistance [31,32,33,34], correlated with the morphological differences observed in CM17-2 (Figure 6). However, exogenous GA_3_ treatment decreased vascular bundle number in CM17-2, suggesting a GA_3_-dependent regulation of vascular patterning in this mutant.

### 2.5. Transcriptomic Analysis of the Maize Dwarf Mutant Under GA_3_ Treatment

#### 2.5.1. Quality Assessment of Total RNA

The quality of total RNA extraction determines the reliability of sequencing data. After assessing the purity, concentration, and integrity of the total RNA, the results showed that the RNA samples had an OD260/280 ratio between 1.9 and 2.1, a concentration ranging from 106 to 358 ng/µL, and RIN values between 9.5 and 10. All RNA samples met the quality requirements for transcriptome sequencing and were suitable for library construction and subsequent sequencing (Supplementary Table S2).

#### 2.5.2. Statistics of Sequencing Data

RNA-Seq sequencing was performed on the quality-passed samples. After sequencing quality control, a total of 116.81 Gb of clean data was obtained, with the percentage of bases having a quality score ≥ 30 (Q30) being no less than 92.83% for all samples (Supplementary Table S3).

#### 2.5.3. Statistics of Reads Aligned to the Reference Genome

The processed data were aligned to the reference genome sequence Zea_mays.Zm_B73_REFERENCE_GRAMENE_4.0.genome.fa using HISAT2 2.0.4 software. The results showed that the number of reads mapped to the reference genome accounted for 94.03–96.63% of the clean reads, while the number of reads mapped to multiple locations in the reference genome accounted for 2.55–3.11% of the clean reads (Supplementary Table S4).

#### 2.5.4. Differential Gene Expression Analysis

**Figure 7:**
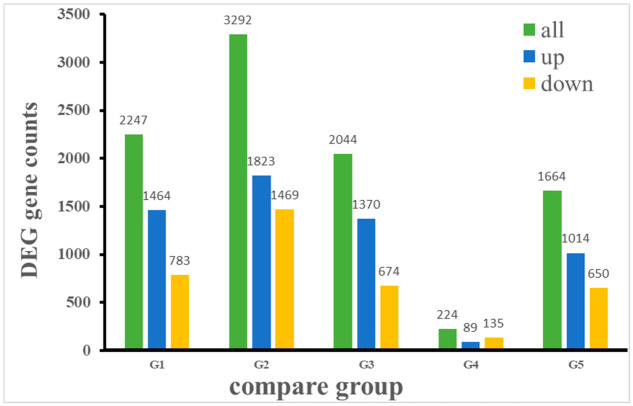
Number of differentially expressed genes in different comparison groups. Note: G1: B73_VS_B73_GA__VS_CM17-2_VS_CM17-2_GA_; G2: B73_VS_CM17-2; G3: B73_GA__VS_CM17-2_GA_; G4: B73_VS_B73_GA_; G5: CM17-2_VS_CM17-2_GA_.

As shown in (Figure 7), the number of differentially expressed genes between the dwarf mutant CM17-2 and the wild-type B73 was counted, and the numbers of genes in each differential expression set were visually displayed using a bar chart. In this chart, green represents all differentially expressed genes, blue represents upregulated genes, and yellow represents downregulated genes. A total of 3292 differentially expressed genes were identified between B73 and the dwarf mutant CM17-2, of which 1823 (55.38%) were upregulated and 1469 (44.62%) were downregulated. A total of 224 differentially expressed genes were identified between B73 and GA_3_-treated B73, of which 89 (39.73%) were upregulated and 135 (60.27%) were downregulated. In contrast, 1664 differentially expressed genes were identified between the dwarf mutant CM17-2 and GA3-treated CM17-2, of which 1014 (60.94%) were upregulated and 650 (39.06%) were downregulated. In addition, a total of 2247 differentially expressed genes were identified when comparing the combined sets of B73 vs. GA_3_-treated B73 and CM17-2 vs. GA_3_-treated CM17-2, of which 1464 (65.15%) were upregulated and 783 (34.85%) were downregulated.

#### 2.5.5. GO Analysis of Differentially Expressed Genes

**Figure 8:**
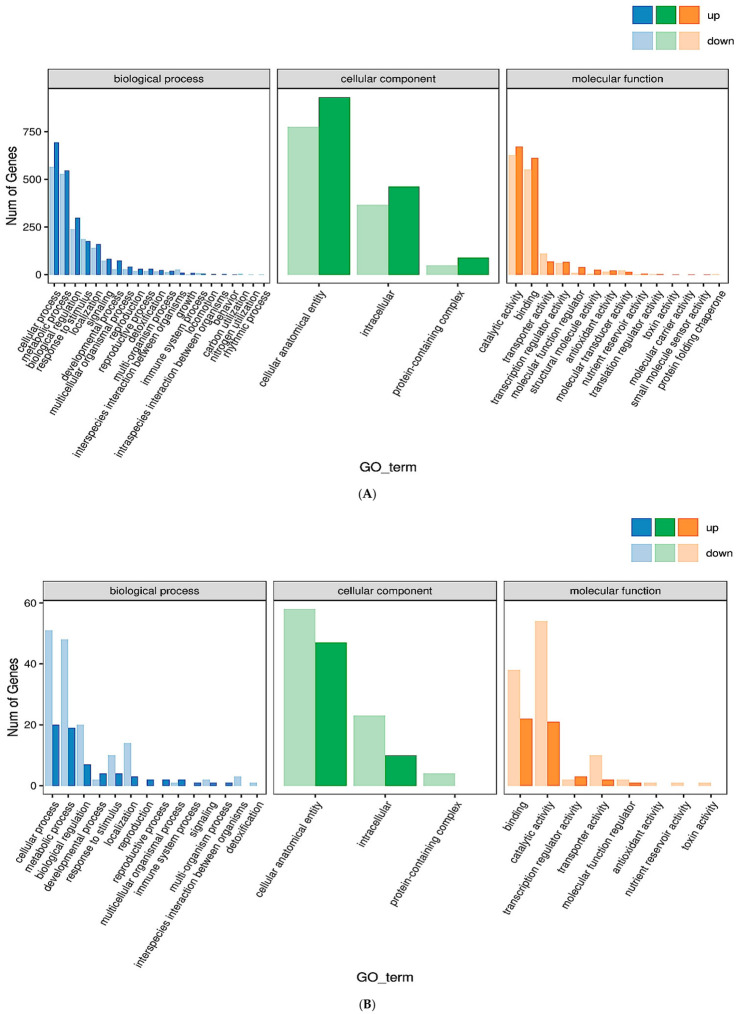

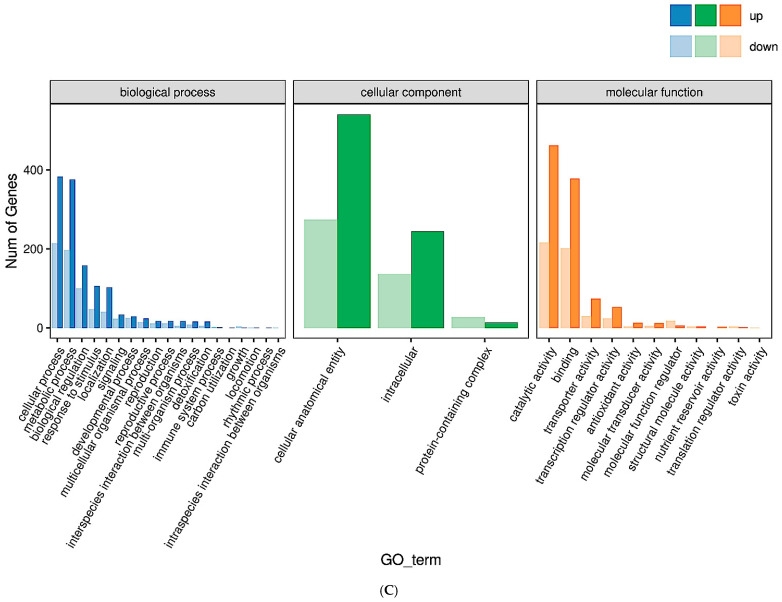
Enrichment of GO function of differentially expressed genes. Note: (**A**): B73_VS_CM17-2; (**B**): B73_VS_B73 GA; (**C**): CM17-2_VS_CM17-2 GA.

GO functional enrichment analysis was performed on the differentially expressed genes between the wild-type B73 and the dwarf mutant CM17-2, covering three main categories: Biological Process (BP), Cellular Component (CC), and Molecular Function (MF). As shown in Figure 8, the differentially expressed genes between wild-type B73 and the dwarf mutant CM17-2 are mainly enriched in the biological processes of cellular process, metabolic process, and biological regulation. The primary cellular components enriched are cellular anatomical entity, followed by intracellular. The main molecular functions enriched are catalytic activity and binding. In summary, the differentially expressed genes identified in this study are predominantly associated with catalytic activity and molecular binding.

#### 2.5.6. KEGG Enrichment Analysis of Differentially Expressed Genes

**Figure 9:**
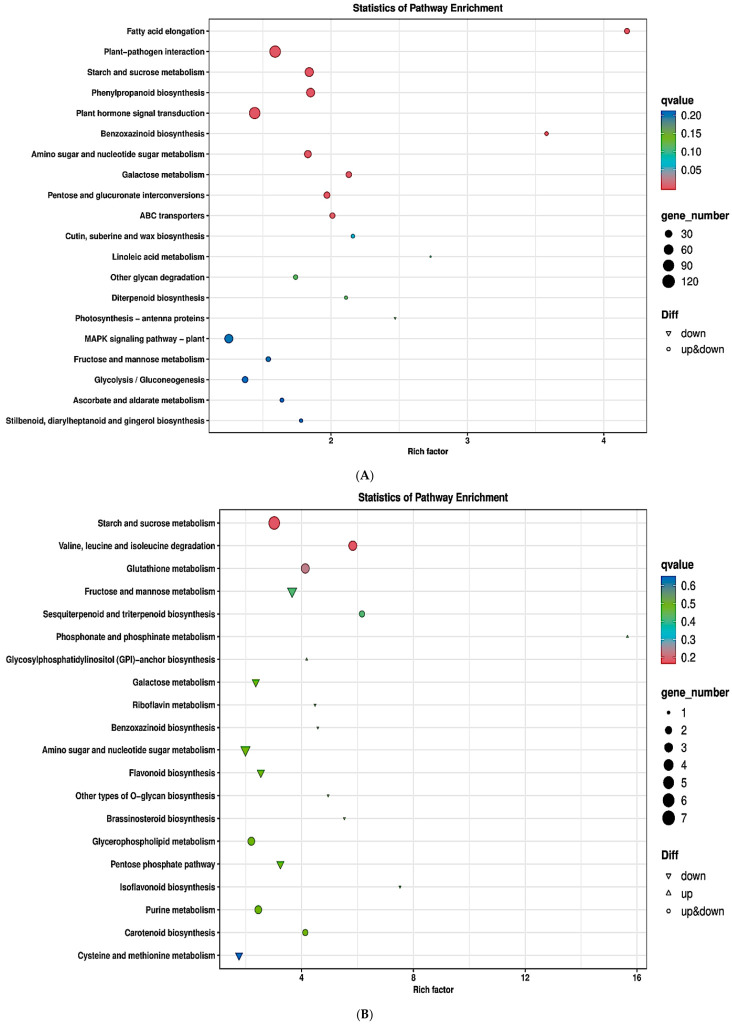

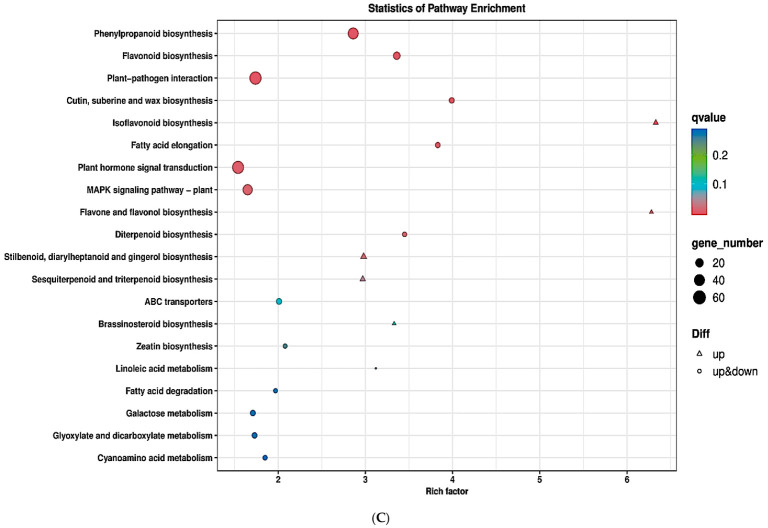
Bubble map of the differentially expressed gene KEGG enrichment. Note: (**A**): B73_VS_CM17-2; (**B**): B73_VS_B73 GA; (**C**): CM17-2_VS_CM17-2 GA.

To further understand the biological functions of the differentially expressed genes, we performed KEGG enrichment analysis on the differentially expressed genes between wild-type B73 and the dwarf mutant CM17-2, with the results shown in (Figure 9). Compared with the wild-type B73, the dwarf mutant CM17-2 exhibited 10 significantly enriched pathways, including fatty acid elongation, plant–pathogen interaction, starch and sucrose metabolism, phenylpropanoid biosynthesis, and plant hormone signal transduction, among others.

Compared with the wild-type B73, GA_3_-treated B73 was primarily enriched in pathways such as starch and sucrose metabolism and valine, leucine, and isoleucine degradation. In contrast, compared with the untreated dwarf mutant CM17-2, GA_3_-treated CM17-2 was mainly enriched in pathways including phenylpropanoid biosynthesis, flavonoid biosynthesis, plant–pathogen interaction, and cutin, suberin, and wax biosynthesis.

#### 2.5.7. Functional Analysis of Differentially Expressed Genes

Using wild-type B73 as the control, differentially expressed genes (DEGs) in the dwarf mutant CM17-2 were identified based on the criteria |log_2_ FC| > 5 and *p* < 0.001. Additionally, DEGs in GA_3_-treated B73 and GA_3_-treated CM17-2 were identified using untreated B73 and CM17-2 as their respective controls. By comparing these DEGs with the candidate genes located within the genomic intervals identified by initial BSA mapping, a final set of 18 candidate genes was selected. The above candidate genes are associated with various biological functions and components, including intracellular organelles of the cell membrane, integral components of membranes, organelles with intracellular membrane structures, response to osmotic stress, cell wall, plant-type cell wall organization, structural molecule activity, membrane constituents, endoplasmic reticulum K20359 PRA1 family protein 1, ATP binding, integral components of membranes, K20359 PRA1 family protein 1, defense response, ADP binding, K15559 Ty1 transposon protein 103 regulator, and magnesium ion binding (Supplementary Table S5).

#### 2.5.8. RT-qPCR Verification

**Figure 10:**
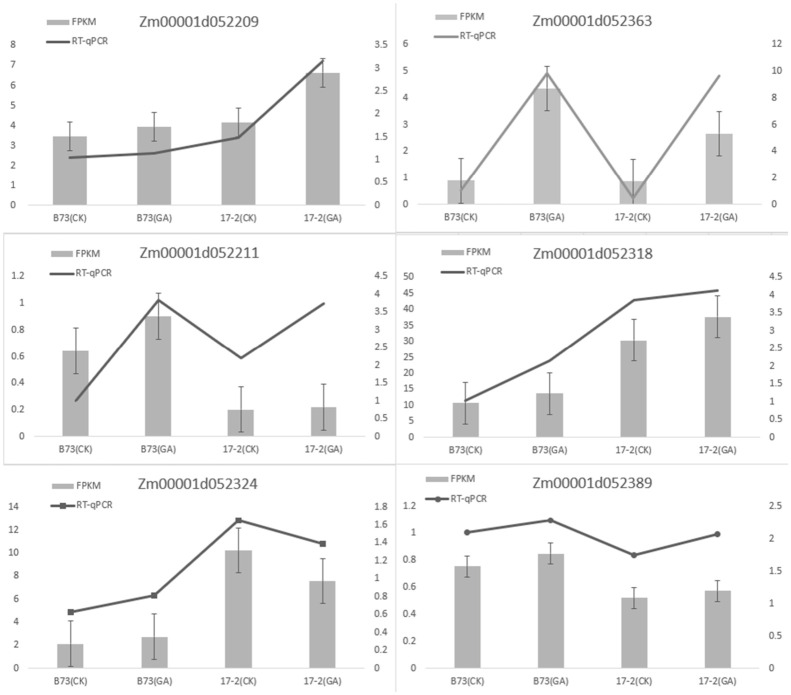
RT-qPCR was performed to verify the expression of six genes. The validation results of the transcriptome data were expressed using both the bar graph (RT-qPCR) and the line graph (RNA-Seq).

To further validate the reliability of the transcriptome sequencing results, this study randomly selected six differentially expressed genes (DEGs) for verification via real-time quantitative polymerase chain reaction (qRT-PCR) (Figure 10). The relative expression levels of these six genes showed consistent trends with the RNA-seq data, fully confirming the reliability of the sequencing results.

#### 2.5.9. Screening of Candidate Genes

Gene annotation was performed for the 18 identified genes (Supplementary Table S6). Based on the annotation information, we filtered out unannotated genes and those with negligible association to the GA pathway, ultimately identifying four potential candidate genes likely associated with dwarfism: Zm00001d052206, Zm00001d052247, Zm00001d052323, and Zm00001d052213.

## 3. Discussion

### 3.1. Phenotypic Characterization of the Maize Dwarf Mutant

Genetic analysis revealed that the dwarf phenotype of CM17-2 is governed by a single recessive locus that suppresses internode elongation, as evidenced by the 3:1 Mendelian segregation ratio observed in the F_2_ population. Phenotypic evaluation demonstrated significant differences between CM17-2 and the wild-type B73, specifically a highly significant reduction in plant height, internode length, and ear height (*p* < 0.01), along with a significant decrease in stem diameter (*p* < 0.05).

Dwarfism entails not merely a reduction in stature but rather an overall optimization of plant architecture. The integration of chemical regulation, such as application of the plant growth regulator EDAH, with specific cultivation practices, including slow-release fertilization, enables the deliberate shaping of an ideal dwarf plant type [35]. Optimized canopy architecture enhances light interception, air circulation, and light transmission within the canopy, thereby increasing grain yield per unit area through improved population productivity [32]. Dwarfing in maize, whether achieved through genetic mutation or chemical intervention, confers advantages in adaptability, stress tolerance, and ultimately yield under specific conditions. Notably, dwarf maize exhibits enhanced drought tolerance. The *ks3-1* mutant displays substantially reduced endogenous gibberellin levels, which directly correlates with its marked reduction in plant height. Under drought stress, the survival rate of the *ks3-1* mutant is significantly higher than that of wild-type B73.

### 3.2. Initial Genetic Mapping of the Maize Dwarf Mutant

Bulk Segregant Analysis (BSA) overcomes key drawbacks of traditional QTL mapping—namely, time-consuming protocols, large mapping intervals, candidate gene identification challenges, and labor intensity—while efficiently distinguishing qualitative from quantitative traits. This approach has emerged as a robust strategy for genetic locus mapping, as demonstrated by its widespread adoption. For instance, Zhu et al. [36] integrated BSA-seq with transcriptome profiling to dissect salt tolerance mechanisms in maize inbred lines AS5 (tolerant) and NX420 (sensitive), identifying two and four overlapping candidate genes, respectively. Similarly, Zhao et al. [37] employed BSA-seq to map 18 shade tolerance-associated genes in soybean across chromosomes 4, 9, and 408. Furthermore, Gao et al. [38] leveraged BSA-seq and RNA-seq to localize three QTLs on chromosomes 1 and 9 in foxtail millet, uncovering nine candidate genes regulating plant height. Collectively, these studies highlight BSA’s efficacy in accelerating candidate gene discovery for complex traits.

BSA-seq analysis preliminarily mapped the candidate locus for the dwarfing trait to chromosomes 2, 4, and 5. Further investigation of the interval on chromosome 4 identified a total of 113 candidate genes. Among these, *Zm00001eb193970* encodes a ubiquitin-protein ligase. Zhao et al. [39] conducted a genome-wide association study (GWAS) on 370 autotetraploid potato accessions and identified ubiquitin-protein ligase as a key candidate gene influencing plant height. Functional analysis revealed that this gene predominantly participates in hormone signaling pathways, including those of gibberellin and brassinosteroid, and acts in concert with other candidate genes—such as *FAR1*, methyltransferases, and ethylene response factors—to collectively modulate plant stature in potato. Sun et al. [40] reported that *GmILPA1*, a subunit of the APC/C E3 ubiquitin ligase complex, exhibits no significant phenotypic difference in plant height compared to the wild type under greenhouse or white light conditions. However, upon exposure to UV-B radiation, the loss-of-function mutant *Gmilpa1* displays a pronounced dwarf phenotype. Mechanistically, UV-B irradiation triggers the accumulation of *GmILPA1* protein, which specifically recognizes and ubiquitinates GmGA2ox-like, a key enzyme in gibberellin catabolism, thereby targeting it for degradation via the 26S proteasome pathway. Furthermore, another E3 ubiquitin ligase, *GmUBL1*, enhances the efficiency of *GmILPA1*-mediated degradation of GmGA2ox-like. The consequent reduction in GmGA2ox-like protein levels attenuates the oxidative inactivation of bioactive gibberellins (e.g., GA1 and GA4). This leads to a relative increase in active GA content, effectively counteracting the inherent inhibitory effects of UV-B radiation on GA levels, thereby maintaining normal plant height under UV-B stress in soybean. *Zm00001eb195440* encodes a WRKY transcription factor, a major class of transcriptional regulatory proteins in plants that are broadly involved in diverse physiological processes, including growth, development, and stress responses [41]. In wild-type rice, gibberellin (GA) promotes the degradation of the DELLA protein SLR1, thereby alleviating growth repression and enabling normal plant height. However, it has been demonstrated that in the *sgsd3* mutant, overexpression of *OsWRKY36* stabilizes *SLR1* through a dual mechanism: first, it directly binds to the promoter of the *SLR1* gene to enhance its transcription; second, it protects the SLR1 protein from GA-mediated degradation. Consequently, both the transcript and protein levels of *SLR1* are aberrantly elevated, leading to sustained suppression of GA signaling, reduced GA sensitivity, and ultimately a semi-dwarf phenotype. Thus, by stabilizing *SLR1*, this WRKY transcription factor functions as a repressor of GA signaling and negatively regulates plant height in rice [42]. Fang et al. [43] identified *ZmWRKY92* as a nuclear-localized protein that possesses transactivation activity. The loss-of-function mutant *wrky92* exhibits a pronounced dwarf phenotype, which is characterized by reduced internode cell size and consequently shortened internodes. Mechanistic dissection revealed that *ZmWRKY92* specifically binds to the W-box elements in the promoter regions of *ZmGA20ox7* (encoding a key enzyme in gibberellin biosynthesis) and *ZmGID1L2* (encoding a GA receptor), thereby activating their transcription. In the *wrky92* mutant, the compromised expression of these two target genes leads to a marked reduction in the level of bioactive GA_3_, which in turn suppresses cell elongation and ultimately results in dwarfism. Collectively, these findings demonstrate that *ZmWRKY92* plays a critical promoting role in the determination of maize plant height by directly orchestrating the expression of genes associated with GA metabolism.

### 3.3. Physiological Responses of the Maize Dwarf Mutant to GA_3_

#### 3.3.1. Phenotypic Analysis of the Effects of Exogenous Substances on the Maize Dwarf Mutant

Gibberellins, auxins, and brassinolide are pivotal regulators of crop plant height. Perturbations in the gibberellin biosynthesis and signaling pathways confer dwarfism in maize. Loss-of-function mutations in biosynthetic or signaling genes disrupt gibberellin homeostasis, resulting in dwarf phenotypes. Conversely, modulating these genes alters bioactive gibberellin levels, thereby influencing plant stature and biomass [44]. Notably, dysregulation of gibberellin metabolism underpins the genetic basis of maize dwarfism, highlighting its central role in developmental plasticity. This study demonstrates that exogenous hormone treatments significantly enhanced seedling length and internode elongation in both the dwarf mutant and wild-type B73, with all measured traits exhibiting statistically significant differences (*p* < 0.05). Notably, GA_3_ treatment elicited the most pronounced promotive effect, alleviating the dwarf phenotype and fully restoring plant height to near-wild-type levels. These results suggest that the dwarf mutant is a gibberellin biosynthesis-deficient mutant. Among the tested hormones (IAA, BL, GA_3_), GA_3_ elicited the most robust response in the mutant. These findings form the basis for subsequent GA_3_-induced transcriptome profiling to decipher the molecular mechanisms governing hormone-responsive phenotypic rescue. Furthermore, gene regulatory network reconstruction will facilitate identification of key regulatory hubs involved in this process, laying the groundwork for breeding lodging-resistant maize cultivars.

#### 3.3.2. Cytological Analysis of Internode Cells in Wild-Type, Dwarf Mutant, and Their Controls Under GA_3_ Treatment in Maize

Elongation of maize stem internodes primarily results from increased cell proliferation and cellular expansion, cytologically. Cytological examination of paraffin-embedded longitudinal sections by Bensen et al. [15] revealed that decreased internode number and shortened cell length primarily underlie the dwarf phenotype of maize mutants. Building on this, Main et al.’s [45] analysis of rice internode cell size demonstrated that elongation is directly determined by cellular dimensions within the elongation zone, influencing plant height and indicating that differential internode lengths drive height variations. Consistent with these findings, our study examined maize stem cell longitudinals and showed that dwarfism reduces longitudinal cell count and length, thereby enhancing lodging resistance. Zheng et al. [46] revealed that vascular bundles, acting as structural support elements, directly determine lodging resistance in maize stems. Robust vascular bundles endowed with adequate strength and resilience enhance wind stress tolerance, thereby mitigating lodging incidence and minimizing associated yield losses. Geng et al. [47] revealed that elevated planting density alters maize stem anatomy, characterized by diminished sclerenchyma thickness, decreased lignified parenchyma cell counts, and reduced vascular bundle area and density. These anatomical deficiencies diminish rind penetration strength and maximum bending moment, contributing to increased lodging incidence under field conditions. Ding et al. [48] revealed that maize stem bending strength is significantly and positively correlated with vascular bundle quantity and size, as well as stem cross-sectional area. These structural features are identified as primary determinants of lodging resistance. In this study, maize stem cross-sectional anatomy was characterized. The dwarf mutant CM17-2 exhibited significantly higher vascular bundle counts than the wild-type B73. Following exogenous GA_3_ application, both CM17-2 and B73 displayed significant vascular bundle count reductions relative to untreated controls.

In this study, maize stem internodes were characterized to elucidate microstructural features. Cellular architecture alterations varied significantly across dwarf germplasm. Plant dwarfism positively correlated with increased cell elongation and proliferation, yet negatively with vascular bundle area and density. While these findings offer mechanistic insights, longitudinal segmentation and multi-sectional analysis are imperative to decipher growth-stage-specific dwarfing mechanisms.

### 3.4. Transcriptomic Analysis of a Maize Dwarf Mutant Under GA_3_ Treatment

The abundance of differentially expressed genes (DEGs) serves as a pivotal indicator for assessing genetic background or physiological status differences across materials [49]. In the comparison between wild-type B73 and dwarf mutant CM17-2, upregulated DEGs significantly outnumbered downregulated DEGs, indicating that the dwarf phenotype is primarily manifested through upregulation of genes. Similarly, in the CM17-2 vs. GA_3_-treated CM17-2 comparison, upregulated DEGs predominated, implying the dwarf mutant’s sensitivity to exogenous GA_3_ and suggesting a potential involvement of hormone signaling pathways in maize dwarfing.

This study reveals that the flavonoid and diterpenoid biosynthetic pathways are specifically and significantly enriched in GA_3_-treated samples compared with the dwarf mutant phenotype. These distinctive pathways may be functionally linked to the altered elongation of maize stem internodes, thereby offering valuable insights for advancing genetic research on maize dwarfism.

In numerous plant dwarfing models, the flavonoid biosynthetic pathway is markedly upregulated, concomitant with increased flavonoid accumulation. Zhou [50] provided direct evidence through grafting experiments that flavonoid biosynthesis plays a critical role in rootstock-induced dwarfing in breadfruit. Grafting breadfruit scions onto *Artocarpus heterophyllus* rootstocks resulted in a marked reduction in plant height, stem diameter, and internode length, yielding a dwarf phenotype exceeding 65%. This dwarfing process was associated with a significant increase in total flavonoid content in the scion stem, accompanied by pronounced upregulation of *AaCHS*, a gene encoding chalcone synthase and a key upstream enzyme in the flavonoid biosynthetic pathway. Correlation analysis further revealed a strong positive relationship between total flavonoid accumulation and *AaCHS* transcript abundance in the scion stem. In contrast, the expression of *AaDFR*, which encodes the bifunctional dihydroflavonol 4-reductase, remained unchanged among scions grafted onto different rootstocks, underscoring the specific regulatory prominence of *AaCHS* in mediating rootstock-induced dwarfing. Collectively, these findings establish a direct mechanistic link between flavonoid biosynthesis and rootstock-conferred dwarfism in breadfruit. Foster et al. [51] conducted comparative transcriptomic analyses between two dwarfing rootstocks (‘M27’ and ‘M9’) and the vigorous rootstock ‘M793’ and identified excessive flavonoid accumulation as a key physiological determinant of dwarfism. The study demonstrated that elevated flavonoid levels in dwarfing rootstocks act in concert with the downregulation of *MdAUX1* and *MdLAX2*, which encode auxin influx carriers, to suppress polar auxin transport. Given that auxin transport is essential for sustaining cell elongation and apical dominance, this disruption directly impairs growth and culminates in reduced tree stature. These findings elucidate a molecular mechanism whereby rootstocks modulate secondary metabolite accumulation—specifically flavonoids—to interfere with hormonal signaling and exert control over scion architecture.

The diterpenoid biosynthetic pathway constitutes a pivotal route for the production of diverse, biologically important secondary metabolites in plants, most notably the gibberellin (GA) phytohormones. Zhang et al. [52] integrated physiological, transcriptomic, and proteomic analyses of the blue fescue dwarf mutant *dw-1* to elucidate the central role of gibberellin (GA) in regulating plant stature. Physiologically, GA levels were markedly reduced in the *dw-1* mutant compared to the wild type, establishing a direct correlation between GA deficiency and the dwarf phenotype. At the molecular level, KEGG enrichment analysis revealed significant enrichment of the diterpenoid biosynthetic pathway—the upstream metabolic route for GA production—at both transcript and protein levels, indicating broad suppression of pathway activity. Collectively, these findings led Zhang et al. [52] to conclude that the downregulation of genes and proteins associated with GA and indole-3-acetic acid biosynthesis constitutes a core mechanism underlying the dwarfism observed in the *dw-1* mutant. Gu et al. [10] performed a comparative analysis of the dwarf mango cultivar ‘Guire 7’ and the standard cultivar ‘Jinhuang’, identifying 4954 differentially expressed genes (DEGs) and 317 differentially accumulated metabolites. KEGG enrichment analysis revealed significant overrepresentation of DEGs in the diterpenoid biosynthesis pathway, whereas differentially accumulated metabolites were enriched in the upstream terpenoid backbone biosynthesis pathway. Despite the enrichment of DEGs in diterpenoid biosynthesis, the dwarf cultivar exhibited markedly reduced accumulation of bioactive gibberellins, including GA_3_ and GA_4_, a phenotype attributed to the upregulation of *GA3ox*. *GA3ox* encodes gibberellin 3β-hydroxylase, a key enzyme that catalyzes the conversion of inactive GA precursors to bioactive GA_3_ and GA_4_. Consequently, the observed upregulation of *GA3ox* paradoxically coincided with diminished levels of active GAs, thereby suppressing plant growth and contributing to the dwarf phenotype. Further transgenic experiments in tobacco demonstrated that exogenous GA application substantially restored normal morphology and stature in GA3ox-overexpressing lines, corroborating the functional role of *GA3ox* in modulating dwarfing traits.

### 3.5. From Membrane Signaling to Wall Mechanics: An Integrated Model for CM17-2 Dwarfism

This study integrates transcriptome-driven candidate gene screening with histological analysis to propose that the dwarfism of CM17-2 results primarily from impaired internode elongation. Histological examination via paraffin sectioning revealed shortened cell length and decreased cell number in mutant internodes. Exogenous GA_3_ treatment significantly rescued plant height, concurrent with enhanced cell elongation, increased cell proliferation, and vascular bundle expansion. Collectively, these findings suggest that CM17-2 retains partial GA responsiveness, implying that dwarfism arises from compromised GA signaling efficacy in internodes and subsequent attenuation of growth-related programs.

At the candidate-gene level, *IP5P*, *CHC1*, and *PRA1/RabR* together with *PRA1K* are all associated with membrane lipid signaling, endocytosis, and vesicle sorting/trafficking. These annotations suggest that disruption of membrane trafficking homeostasis may reduce the localization and turnover efficiency of GA signaling components, thereby lowering GA sensitivity and diminishing signaling output in internode tissues [53,54,55,56]. The cell wall loosening and remodeling module—represented by *EXPA*, *QUA2/PMT*, and xyloglucan O-acetyltransferase—provides a direct molecular explanation for shortened cells [57,58]: when GA signaling output is insufficient or cell wall modification is constrained, wall plasticity decreases and cells fail to expand longitudinally, ultimately leading to internode shortening [53,57,59]. In parallel, *PRP22* (an RNA helicase implicated in pre-mRNA processing/splicing), together with genes involved in metabolic supply and small-molecule homeostasis (*SK*, *HIBADH*, *UGT85A*, *UGT-DLGT*, and a PPTase-related protein), suggests that post-transcriptional processing capacity, metabolic support, and glycosylation-mediated regulation may jointly limit cell proliferation and tissue growth, consistent with the reduced cell number observed in the mutant [60,61]. A defense/stress branch (*TTL1*, *RPP13*, and *PPH*) may further suppress elongation growth through a growth–defense trade-off, thereby amplifying the dwarf phenotype [60,62].

GA_3_ application may compensate for these constraints by elevating GA signaling output. On the one hand, it can reactivate cell wall loosening/remodeling processes, promoting cell elongation and resulting in longer cells; on the other hand, it may restore growth-related expression programs and metabolic support, enhancing cell division, increasing cell number, and promoting internode elongation. The enhanced vascular bundles observed in transverse sections further imply that tissue differentiation capacity increases alongside GA signaling; notably, the cell wall reinforcement/lignification module represented by *LAC23* (laccase-23) may contribute to vascular bundle maturation and structural reinforcement, consistent with the more developed vascular phenotype [63,64].

**Figure 11:**
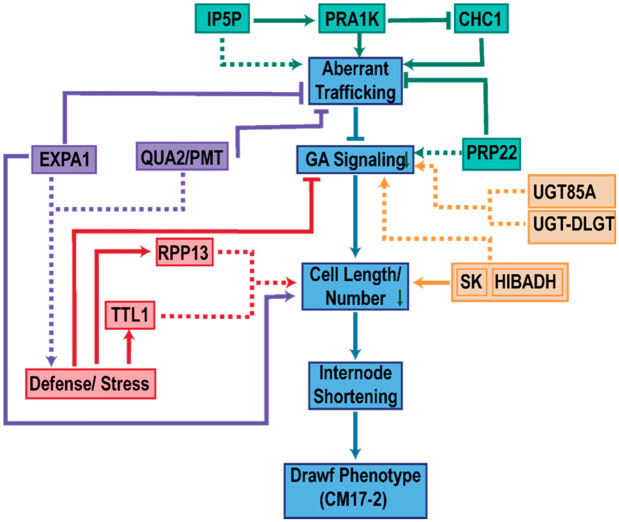
An Integrated Model for CM17-2 Dwarfism. Note: Solid lines: Major regulatory pathways constructed based on functional annotations and phenotypic/histological results. Dashed lines: Indicate putative indirect relationships. Green module: Genes related to lipid signaling and vesicle transport/endocytosis. Purple module: Genes associated with cell wall loosening and remodeling. Red module: Stress/defense-related module and its representative genes. Orange module: Genes involved in metabolic supply, small molecule homeostasis, and glycosylation regulation. Blue module: Core growth output and phenotypic nodes.

Overall, we propose a working model of “perturbed membrane trafficking → reduced effective GA signaling output → insufficient cell wall remodeling and cell proliferation → internode shortening,” (Figure 11) and suggest that GA_3_ promotes coordinated recovery of cell elongation, cell proliferation, and vascular development by increasing GA signaling output. Given the current lack of gene-level directional evidence and causal validation, future work should test key nodes using qPCR, GA-response readouts (e.g., *DELLA* dynamics), and genetic interaction/functional assays to refine and validate this model.

### 3.6. Candidate Gene Analysis

Gene annotation of the 18 differentially expressed genes revealed four potential candidate genes likely associated with dwarfism (Supplementary Table S12).

*Zm00001d052323* encodes a brassinosteroid LRR receptor kinase, which may inhibit BR signaling [65]. BR-insensitive mutants have been observed to exhibit impaired GA signaling perception, whereas certain GA-hypersensitive mutants display enhanced sensitivity to BRs [66]. Furthermore, Arabidopsis mutants with defective BR signaling show severe impairment in bioactive GA production, correlating with the expression of defective GA biosynthesis genes [67]. Consequently, we hypothesize that a deficiency in BR signaling leads to severe dwarfism, often accompanied by obstructed GA signaling.

*Zm00001d052206* encodes Protein EXORDIUM (*EXO*). *EXO* does not directly regulate BR levels or sensitivity; rather, it coordinates BR responses with environmental or developmental signals to ensure effective BR-promoted leaf cell expansion [68]. In the absence of *EXO*, BRs fail to properly activate growth-related genes (e.g., *WAK1*, *EXP5*, *KCS1*), thereby attenuating BR-induced growth [68]. Elevated cytokinin levels reduce *EXO* expression, impairing BR signal transduction and resulting in plant dwarfism [69].

*Zm00001d052247* encodes shikimate kinase 2, a key enzyme in the shikimate pathway. This pathway is essential for synthesizing aromatic amino acids (phenylalanine, tyrosine, and tryptophan) and provides precursors for secondary metabolites, including flavonoids, lignin, and plant hormones [70,71,72]. Tryptophan, a downstream product of this pathway, serves as a precursor for IAA biosynthesis. Deficiency in this key enzyme leads to reduced auxin (IAA) levels and increased jasmonic acid (JA) accumulation. This hormonal antagonism inhibits cell division and elongation, causing dwarfism [71].

*Zm00001d052213* encodes the pectin methyltransferase *QUA2*. *QUA2* is a Golgi-localized protein in *Arabidopsis* essential for the synthesis of homogalacturonan (HG), a major component of pectin [73]. Studies indicate that *qua2* mutants (e.g., *qua2-1*) exhibit reduced cell adhesion and approximately 50% lower HG content in cell walls compared to wild-type plants [73]. As a key factor in maintaining normal pectin synthesis, the loss of *QUA2* function causes a sharp reduction in HG content and cell wall integrity damage. Plants respond to this damage by activating BR signaling and upregulating negative regulators such as *AtPRX71*, establishing a growth-restricting feedback mechanism that ultimately manifests as dwarfism [73,74,75].

## 4. Materials and Methods

### 4.1. Materials

The dwarf mutant CM17-2, derived from B73 via carbon ion beam mutagenesis, was planted at the experimental base of Gansu Agricultural University. CM17-2 (male parent) was crossed with B73 (female parent) to generate the hybrid F_1_ generation. The following year, the F_1_ plants were grown at the same experimental base and self-pollinated to construct the F_2_ population.

### 4.2. Methods

#### 4.2.1. Phenotypic Measurements of the Dwarf Mutant CM17-2 and Wild-Type B73 Plants in the Field

Three biological replicates each of the dwarf mutant CM17-2 and wild-type B73 plants were selected for phenotypic measurements: plant height, internode length, and ear height were measured using a ruler, and stem diameter was measured using a vernier caliper.

#### 4.2.2. Genetic Analysis of the Dwarf Mutant in Maize

The dwarf mutant CM17-2 was crossed with the inbred line B73 (wild type) to generate the F_1_ generation. The F_1_ hybrids were then self-pollinated to produce the F_2_ generation. The numbers of tall and dwarf plants in the F_2_ segregating population were recorded, and a chi-square test was used to verify the inheritance pattern of the mutant trait.

#### 4.2.3. Selection and Sampling of Extreme Phenotypic Plants, DNA Extraction, and BSA (Bulked Segregant Analysis) Sequencing

At least 220 plants of the CM17-2 F_2_ population were grown. At the three-leaf-one-heart stage, segregation for plant height in the F_2_ population was observed. Plants exhibiting a 3:1 tall-to-dwarf segregation ratio were selected. In a laminar flow hood, internode tissues from 50 extremely tall and 50 extremely dwarf plants were collected separately, placed into 5 mL cryovials, immediately frozen in liquid nitrogen, and stored at −80 °C for subsequent use. Genomic DNA was extracted using the CTAB method, following the protocol described by Saghai-Maroof et al. [76]. The DNA samples were sent to Annoroad Gene Technology (Beijing, China) Co., Ltd. for library preparation and deep DNA sequencing. The quality and concentration of the DNA were assessed prior to sequencing. Two extreme bulked pools were constructed separately—each consisting of equimolar DNA from 50 tall plants and 50 dwarf plants of the CM17-2 F_2_ population, respectively. Paired-end sequencing was performed on the BGI T7 high-throughput sequencing platform with a sequencing depth of 20×. Data analysis was performed using a Linux server and the R programming language, following the analytical methods described in the study by Ochar Kingsley et al. [77].

#### 4.2.4. Exogenous Hormone Treatment

Seeds of uniform size and fullness were selected from the F_2_ population derived from a cross between wild-type B73 and the dwarf mutant CM17-2. The seeds were surface-sterilized with 0.5% NaClO for 10 min, rinsed three to five times with double-distilled water (ddH_2_O), and imbibed in distilled water for 12 h. Excess surface moisture was removed using sterile filter paper. Seeds were sown in pots (10 cm diameter × 10 cm height) containing a sterile mixture of vermiculite and distilled water (5 g:1 mL, *w*/*v*). Ten seeds were sown per pot and irrigated with 50 mL of distilled water every two days. Plants were grown in a controlled-environment chamber set at 25 °C/22 °C (day/night) with a 14 h/10 h (light/dark) photoperiod, 60% relative humidity, and a photosynthetic photon flux density (PPFD) of 600 μmol·m^−2^·s^−1^.

When distinct segregation in plant height was observed in the F_2_ population, seedlings were subjected to eight treatments with three biological replicates each: CK_B73_: distilled water, CK_B73_: distilled water; IAA_B73_: 100 μmol/L IAA; GA_B73_: 100 μmol/L GA; BL_B73_: 0.83 μmol/L BL; CK_CM17-2_: distilled water; IAA_CM17-2_: 100 μmol/L IAA; GA_CM17-2_: 100 μmol/L GA; BL_CM17-2_: 0.83 μmol/L BL. Except for the control (CK), which was quantitatively irrigated with 50 mL of distilled water every two days, all other treatments received 50 mL of the respective hormone solutions at the same interval. Upon the appearance of distinct phenotypic differences between the treatments and the control, the first internodes were harvested. For each treatment, three biological replicates were immediately snap-frozen in liquid nitrogen for 3–4 h and subsequently stored at −80 °C for future analysis. For the eight treatments mentioned above, the optimal concentrations were selected based on preliminary screening in our laboratory: 100 μmol/L for IAA, 100 μmol/L for GA_3_, and 0.83 μmol/L for BL (No relevant papers have appeared in print to date). Relevant physiological indices were measured upon the appearance of distinct differences between the treatments and the control.

#### 4.2.5. Cytological Observation

Cytological observation was performed using the paraffin sectioning method, following the experimental procedure described by Pan Haoyu et al. [78].

#### 4.2.6. RNA Extraction, Library Preparation, and Transcriptome Sequencing

After passing quality control checks, RNA libraries were constructed following the protocol described by Meng et al. [79]. Once the libraries passed quality assessment, paired-end sequencing (PE150) was performed using the Illumina NovaSeq 6000 platform (Illumina, Inc., San Diego, CA, USA).

#### 4.2.7. Transcriptome Analysis

After the sequencing run was completed, the raw data were analyzed using the bioinformatics pipeline provided by BMK Cloud, the cloud platform of Biomarker Technologies Corporation (Beijing, China). The raw sequencing data were first filtered to obtain clean reads, which were then aligned to a designated reference genome to generate mapped data. Subsequent analyses included library quality assessment, structural-level analysis, differential expression analysis, gene functional annotation, and functional enrichment. Furthermore, the BMK Cloud platform enabled in-depth data mining and a suite of interactive, customizable transcriptome analyses, such as gene retrieval and visualization, identification of unique and shared genes, screening of differentially expressed genes, and gene set enrichment analyses (e.g., GO and KEGG).

#### 4.2.8. DEG Real-Time Fluorescence Quantitative PCR (RT-qPCR) Validation

Reverse transcription and real-time quantitative PCR were performed using the Evo M-MLV Reverse Transcription Kit from Accurate Biotechnology (Changsha, China) and the SYBR Green Pro Taq HS Premixed qPCR Kit (with ROX) (Accurate Biotechnology (Hunan) Co., Ltd., Changsha, China). The reverse transcription conditions were set as follows: 37 °C for 15 min, 85 °C for 5 s, and hold at 4 °C. Primers were designed (Supplementary Table S6) and RT-qPCR was performed on the QuantStudio5 real-time PCR system. The relative expression levels of the selected genes were calculated using the 2^−∆∆CT^ method, with three biological replicates per treatment [80].

## 5. Conclusions

In this study, an F_1_ hybrid was generated by crossing the dwarf mutant CM17-2 with wild-type B73, followed by self-pollination to produce an F_2_ population. Preliminary investigations were conducted through phenotypic characterization of the mutant, genetic analysis, phenotypic evaluation of the F_2_ population, and initial mapping of the mutant gene. The main conclusions are as follows:

1. Genetic analysis revealed that the dwarf phenotype in the mutant CM17-2 is controlled by a single recessive gene.
2. Treatment of the dwarf mutant with exogenous hormones BL, IAA, and GA_3_ revealed that exogenous GA_3_ elicited a more pronounced response in promoting stem elongation in dwarf maize seedlings.
3. BSA sequencing analysis of the F_2_ segregating population of the dwarf mutant CM17-2 preliminarily identified candidate genes located on chromosomes 2, 4, and 5.
4. Using RNA-Seq technology, we analyzed differentially expressed genes (DEGs) between wild-type B73 and the dwarf mutant CM17-2, as well as between GA_3_-treated CM17-2 and its untreated control. The DEGs were primarily enriched in the following biological processes: cellular processes, metabolic processes, and biological regulation. The main enriched cellular components included cellular anatomical entities and intracellular components. In terms of molecular function, the DEGs were predominantly associated with catalytic activity and molecular binding. Key significantly enriched KEGG pathways included fatty acid elongation, plant–pathogen interaction, starch and sucrose metabolism, phenylpropanoid biosynthesis, and plant hormone signal transduction.
5. In this study, we integrated Bulked Segregant Analysis (BSA) and RNA-Seq data from an F_2_ population generated by crossing the dwarf mutant CM17-2 with wild-type B73. This analysis initially delineated 18 genes on chromosome 4. Following functional annotation, we excluded unannotated loci and those unrelated to the GA pathway, ultimately identifying four potential candidate genes likely associated with dwarfism. These findings lay a theoretical foundation for the functional characterization and molecular cloning of dwarfism-related genes in maize.

## Figures and Tables

**Table 1:** Results of F_2_ population separation ratio statistics.

Materials	Normal	Dwarf	Normal/Dwarf	X2, *p*-Value
CM17-2	221	71	3.11	0.073, 0.787

**Table 2:** Measurements of plant height, internode height, spike height and stem thickness in.

Material	Plant Height/cm	Internode Height/cm	Spike Height/cm	Stem Thickness/mm
B73	252.50 ± 2.18	8.20 ± 0.30	107.00 ± 1.80	23.07 ± 0.46
CM17-2	148.08 ± 0.38 **	13.13 ± 0.32 **	59.67 ± 1.61 **	24.88 ± 0.29 *

Note: Mean ± standard deviation; * represents significant differences at the *p* < 0.05 level; ** represents significant differences at the and *p* < 0.01 levels.

## Data Availability

The original contributions presented in the study are included in the article/Supplementary Materials. The data presented in the study are deposited in the NCBI Bio Project repository, accession number PRJNA1462160. Further inquiries can be directed to the corresponding authors.

## Supplementary Materials

The following supporting information can be downloaded at: https://www.mdpi.com/article/10.3390/plants15111715/s1, Table S1. Screening and annotation of candidate genes; Table S2. Quality control of total RNA for RNA-Seq; Table S3. Statistical results of sequencing data; Table S4. Comparative statistics; Table S5. Functional analysis of differential genes between wild-type B73 and mutants; Table S6. Primer sequences for real-time quantitative PCR (RT-qPCR); Table S7. Gene annotation of candidate genes; Table S8. Expression profiles of 18 candidate genes; Table S9. DGEs involved in plant hormone signal transduction; Table S10. DGEs involved in the diterpenoid pathway; Table S11. All differentially expressed genes; Table S12. Expression profiles of four potential candidate genes likely associated with dwarfism.
